# Memphis Medical College

**Published:** 1849-10

**Authors:** 


### MEMPHIS MEDICAL COLLEGE.

It has been publicly announced by some of the professors in the Memphis Medical School that its organization has been broken up and its operations for the present suspended. This is a catastrophe for which the public could hardly have been prepared. With so bright a promise as the institution gave a year ago it was not to be expected that it would meet with so sudden an eclipse. But there is much else in our changeful world besides the "spring of love" to remind us of

> "The uncertain glories of an April day,
> Which now shows all the beauties of the sun,
> And by and by a cloud takes all away."

